# Incremental high pressure torsion as a novel severe plastic deformation process: Processing features and application to copper

**DOI:** 10.1016/j.msea.2014.12.041

**Published:** 2015-02-25

**Authors:** A. Hohenwarter

**Affiliations:** Department of Materials Physics, Montanuniversität Leoben and Erich Schmid Institute of Materials Science, Austrian Academy of Sciences, Jahnstr. 12, A-8700 Leoben, Austria

**Keywords:** High-pressure torsion, Incremental, Severe plastic deformation (SPD), Upscale, Ultrafine-grained, Copper

## Abstract

High pressure torsion is known as one of the most popular severe plastic deformation processes. However, it has certain size limitations, especially regarding the thickness of the processed samples. In this contribution incremental high pressure torsion is introduced as a novel severe plastic deformation process. This further development of conventional high pressure torsion is capable of delivering specimens having an extraordinarily high aspect-ratio of thickness to diameter. The features of this process combined with a case-study on a pure copper specimen with a deformed diameter of 50 mm and a thickness of 40 mm is presented.

## 1 Introduction

Among the huge variety of severe plastic deformation (SPD) techniques available nowadays, high pressure torsion (HPT) represents a technique with some remarkable advantages. As an important feature it involves considerably high amounts of hydrostatic pressure which inhibits crack-formation during deformation and leads so to almost no limitation in the applicable strain. Consequently, this technique also allows SPD of intermediate and high strength starting materials, such as steels and tungsten [1,2] and this even at low homologous temperatures. Furthermore, HPT is also fairly simple making the method quite reliable and cost-effective compared to other SPD processes. These reasons may contribute to the still rising interest in this technique in the SPD-community which is reflected by the abundance of published papers, see for example [1–6] and conference contributions dealing with HPT processed materials, for example presented at *NanoSPD6* in Metz, France.

Although this technique should theoretically deliver specimens with strongly inhomogenous microstructures, homogeneity in terms of hardness can be reached along the radius. The reason is the existence of a lower limit of grain-fragmentation after applying a material and processing parameter dependent saturation strain. This leads to the minimum grain size and thereby the highest hardness and strength at low disk radii after employing an appropriate minimum number of rotations. This saturation behavior is an important requirement for achieving large scaled specimens and products with satisfyingly homogenous hardness distributions. Nevertheless, an undeniable disadvantage of HPT has always been the small specimen size. In order to overcome the status and reputation of HPT as an academic curiosity, much effort has been taken over the last years to upscale the process and to increase the sample size, see e.g. [7]. For a long time samples were restricted to samples sizes around 10 mm in diameter. Later diameters of 30 mm and 7 mm in thickness were presented [8]. However, it was also shown that the enlarged specimens also contain a certain axial inhomogeneity, more specifically an inhomogeneity across the thickness of the specimens may occur when the aspect ratio of thickness, *t*, to diameter, *d*, shortly (*t*/*d)* of the HPT disk is too large [9]. This is a consequence of excessive strain localization which occurs by choosing a too large specimen aspect ratio which is amplified when the metal or alloy shows a high propensity for deformation localization, such as pure magnesium and various Al-alloys. From the technical viewpoint the diameter of HPT specimens, keeping some basic design criteria in mind, can be enlarged with moderate effort, however, the problem of the axial inhomogeneity restricts the sample size in one dimension drastically. In order to overcome this problem a further development of high pressure torsion, which will be called incremental high pressure torsion (IHPT), is proposed. It should allow the production of large specimens in all three dimensions with an ultrafine or nanocrystalline microstructure.

This technical extension may increase the application possibilities of materials processed by HPT such as for medical [10] or electronic applications [11] for which other SPD-processes have been so far favoured [12,13]. The process itself and the technical features combined with a case study on pure copper will be presented in this work.

## 2 Experimental

For the demonstration of IHPT technically pure copper (99.9%) was used. The specimens used for the deformation process had the shape of a cylinder with a total height of 70 mm and a diameter of 50 mm. Before processing the samples were annealed at 700 °C for 1 h, in order to obtain a fully recrystallized starting microstructure that can be used to distinguish between deformed and un-deformed areas. For that purpose the deformed specimens were additionally etched using a commercially available etching detergent for copper. The IHPT process that will be explained in detail in the following section, was carried out on a hydraulic machine with a load of approximately 400 t. The rotational speed was approximately 0.07 rotations per minute. A quantitative evaluation of the changes in the mechanical properties due to IHPT after deformation was performed with Vickers micro-hardness measurements using a microhardness tester from Buehler (Micromet 5104). The indents were carried out with a load of 300 gf and dwell times of 15 s. The positioning of the individual indents will be explained in conjunction with cross-sections of the deformed specimen. The microstructural investigation was performed with a light microscope from Olympus (BX51) and a scanning electron microscope (Zeiss 1525) using back scatter electron contrast.

## 3 Results

### 3.1 Processing features

**Fig. 1:**
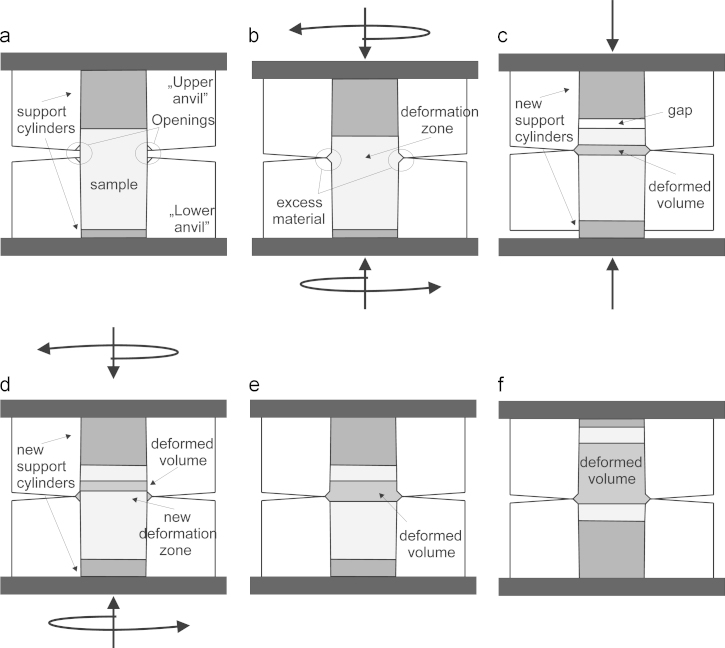
Schematic of incremental high pressure torsion (IHPT) illustrating some significant processing steps. (a) The unloaded setup. (b) Anvils with loaded specimen ready for shear deformation. (c) Shift of deformed material through change of the support cylinders. (d) Next deformation step. (e) Deformed volume after second deformation step. (f) Final stage of deformed specimen after several deformation steps.

**Fig. 2:**
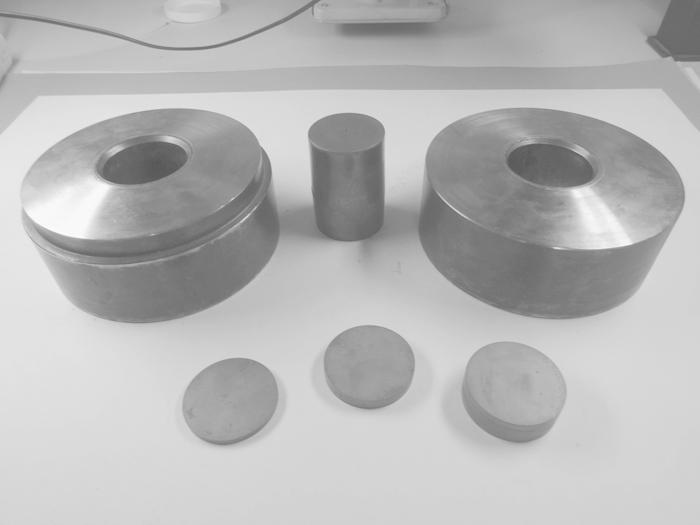
Representation of the tool used for IHPT consisting of two anvils in the back and various disks stacked together to different thicknesses in the front acting as support cylinders and the cylindrical specimen in the middle.

The most important processing steps of IHPT are presented in Fig. 1. Very similar to HPT the specimen in IHPT is deformed between two anvils which are loaded with pressures in the range of several gigapascals. Here, however, the anvils have a drilling hole through the anvils. The inner hole of the anvils are slightly conical, see Fig. 1a, so that the diameter of the hole is somewhat larger at the top of each anvil than on the bottom. The cylinder-like specimen is inserted into the anvils and the local position of the specimen within the anvils is confined by the height of two additional cylinders, named support cylinders, inserted from the outside. In the unloaded state the slight conical shape of the upper drilling hole prevents the upper anvil from contacting the lower anvil. This is because the specimen can only move into the upper anvil as far as the diameter of the specimen is smaller than the inner diameter of the anvil, which becomes smaller the further the specimens moves into the anvil. At the top of each anvil there is also a small opening to concentrate the later ongoing shear deformation in the deformation zone and this opening also ensures that excess material can flow out as it is the case in classical HPT-anvils. When the specimen is loaded, the entire specimen becomes also slightly conical according to the shape of the anvils and the freestanding material between the anvils bulges, gets compressed and the excess material flows out at the openings as illustrated in Fig. 1b. A further displacement of the specimen inside the anvils is suppressed by the support cylinders. The excess material is confined between the anvils and generates the hydrostatic pressure component due to the evolving friction and also prevents the anvils from touching and causing excessive wear. Then, one anvil is rotated against the other, Fig. 1b. The presence of the compressive force combined with the conical shape of anvil and specimen induces tangential friction forces which grip the specimen in the anvils and allows a free shear deformation only in the deformation zone during the process. The deformation state is at this stage very similar to conventional HPT. After a certain number of rotations the specimen is unloaded. Then a different pair of support cylinders is inserted, where the lower one has a larger height, in this case 4 mm, and the top support cylinder is for the same amount thinner, namely 4 mm. Because of this, there is a small gap between the support cylinder and the specimen, see Fig. 1c. When the pressure is applied again the lower support cylinder pushes the specimen upwards to fill the gap. In the same moment the upper and the lower anvil move together downwards and new undeformed material moves into the deformation zone, see Fig. 1d. However, the excess material from the first deformation step cannot move and remains in the openings. Then, one anvil is again rotated against the other for a certain number of rotations, Fig. 1d, and the new undeformed material located in the deformation zone gets deformed. Due to this incremental process the deformed volume increases, Fig. 1e. By doing this process multiple times and by adequately changing the dimensions of the support cylinders the volume of the deformed material increases continuously, Fig. 1f. The growth of the deformed material zone is controlled by the height change of the support cylinders between consecutive deformation steps and defines a certain step size. This step should be smaller than the thickness of the deformation zone evolving in one deformation step to ensure a distinctive overlap of the deformation zones between following deformation steps. The support cylinders can also be replaced with disks, which can be stacked together to a certain height and therefore, a large number of different individually manufactured support cylinders can be avoided. In Fig. 2 the main parts of the deformation process can be seen. In the back, the two anvils made from a hardened steel are presented and in between a typical specimen with a diameter of 50 mm is visible. In the front, disks stacked together to various heights are shown, which act as the support cylinders.

### 3.2 Application of IHPT to copper

In order to demonstrate the feasibility of this technique a case study on pure copper is presented. For the study technically pure copper (99.9%) was used and two different specimens were produced. The first specimen was subjected to 15 rotations to induce a high amount of shear deformation that should allow for a saturated microstructure along the radius. The second specimen was deformed using the IHPT-principle. At first it was deformed to 15 rotations again as the first specimen. Then, the deformation zone was changed in the axial direction by shifting the deformation zone in the tool and another 15 rotations were applied afterwards. In total this incremental process was repeated nine times or in other words 9 deformation steps were applied. In this way the deformed volume increased with every single step where within one step 15 rotations were applied. During one deformation step the deformation zone had a thickness of approximately 8 mm. The step size between following deformation increments was 4 mm. In this way an overlap of the deformation zones between consecutive deformation steps was guaranteed. As support cylinders disks with a thickness of 4 mm were in use and stacked together to get the appropriate heights, see also the examples in Fig. 2.

**Fig. 3:**
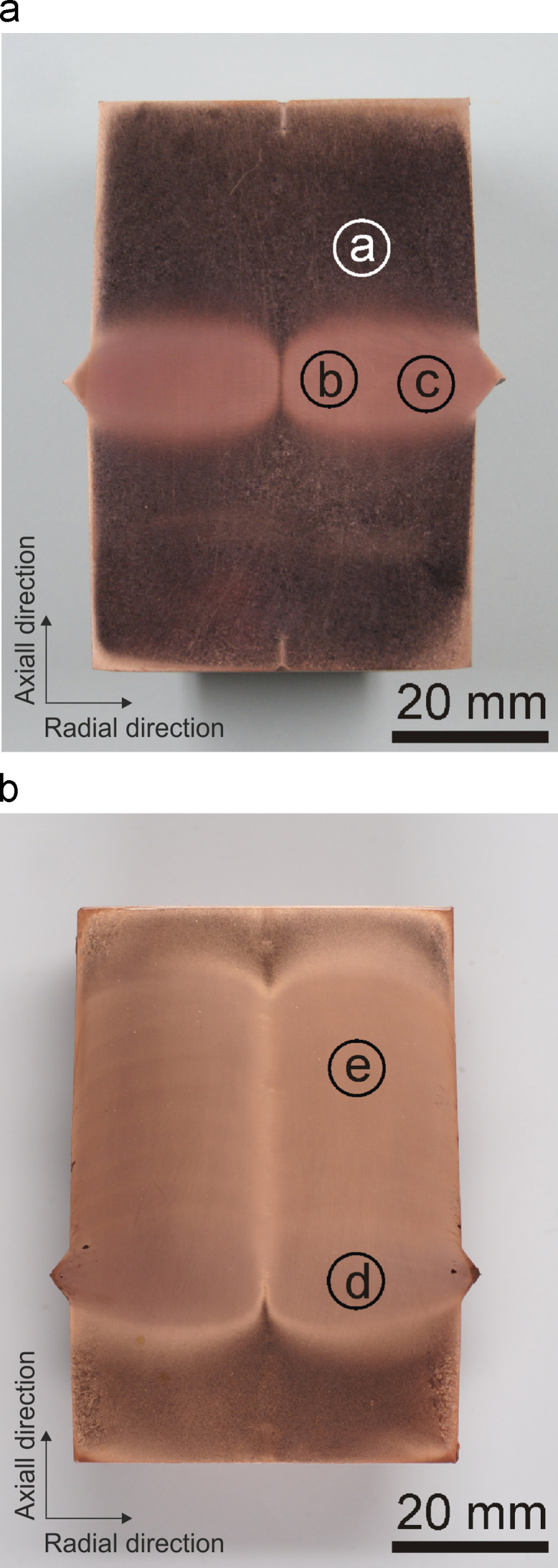
Micrographs of the deformation specimens where the deformed areas remain less etched compared to the undeformed one. (a) Entire specimen cut in half after one deformation step. (b) Second specimen after nine deformation steps.

Photographs of the specimens subjected to IHPT are presented in Fig. 3, which represent the plane cut through the center of the cylinders that were additionally etched to clearly differentiate the deformed from the un-deformed areas. Fig. 3a shows an overview picture of the first specimen deformed with 15 rotations. In this case the deformation zone was chosen to be in the middle of the specimen. The deformation zone is clearly visible and shows a different contrast compared to the top and bottom region where the specimen did not experience a shear deformation. In the middle of the deformation zone a slightly brighter line can be seen which refers to a lower deformed region in the center, where theoretically no deformation can occur. In Fig. 3b the second processed specimen after 9 deformation steps, in which every deformation step consisted of 15 rotations, is shown. The deformed volume has largely increased due to the consecutive changes of the deformation zone in the axial direction. At the bottom and the top of the specimen there is still an undeformed area visible. The intention here is to clearly show the deformation zone. It can be avoided by performing the first deformation step at one end of the specimen and performing as many deformation steps as required to reach the other end of the specimen.

**Fig. 4:**
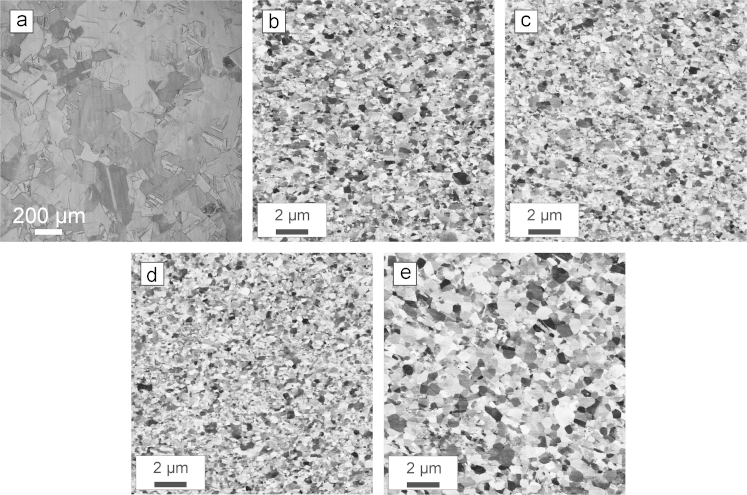
Microstructural observations of areas indicated in Fig. 2. (a) Undeformed microstructure. (b–d) Examples of ufg-microstructures. (e) Slightly annealed structure taken from the area deformed in the first deformation step.

In order to investigate the microstructure more in detail a series of micrographs using light microscopy and scanning electron microscopy is presented in Fig. 4. The micrographs are taken from regions a–e which are indicated in Fig. 3. In Fig. 4a the undeformed recrystallized structure is shown as a reference. Fig. 4b and c represents areas at different radii away from the deformation axis after one single deformation step. The deformed structure is well-refined into the ultrafine-grained regime and looks very similar. In Fig. 4d and e microstructures from the specimen deformed to 9 deformation steps are shown. Fig. 4d originates from an area, which was deformed in the last deformation step and Fig. 4e represents a section, which was deformed in the first deformation step. A difference in the microstructural size can be noticed, which will be discussed later in more detail.

**Fig. 5:**
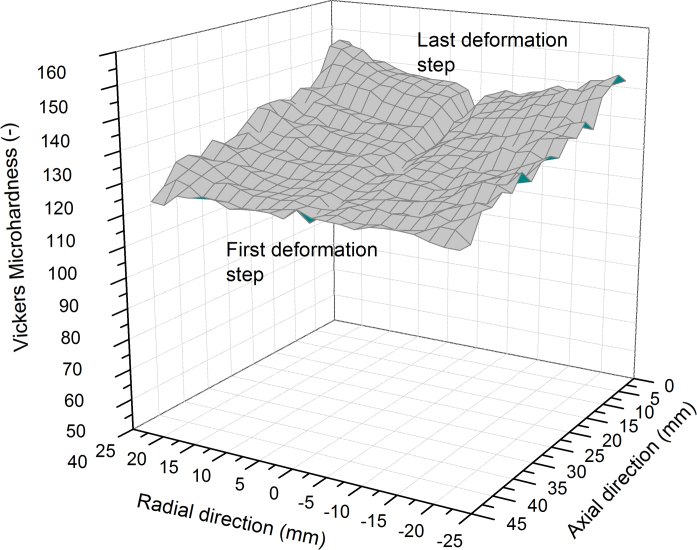
Hardness map along the radius and the axial direction measured for the specimen deformed up to nine deformation steps.

A quantitative evaluation of the change in the mechanical properties due to IHPT after deformation were performed with micro-hardness measurements. The indents were carried out on the metallographic cut of the specimen deformed with 9 deformations steps, as presented in Fig. 3b. The load was 300 gf and the indents were placed on a rectangular grid with a step size of 2 mm on the deformed areas of the specimen in the radial and axial direction,^1^ respectively. The hardness level of the recrystallized structure was approximately 45 HV. In Fig. 5 the hardness map is presented. As shown, the hardness along the radius is at a reasonably constant level. However, in the axial direction there is a gradient in the hardness. The highest hardness levels along the radial direction (~140 HV) are reached in the area which was deformed in the last deformation step and the lowest hardness (~120 HV) is present in the region which was deformed in the first deformation step. This is also consistent with the microstructural observations, which imply a slightly larger grain size for areas which were deformed at the beginning of the process, see Fig. 4e, compared to areas which experienced the deformation in the later deformation steps, Fig. 4d.

## 4 Discussion and summary

**Fig. 6:**
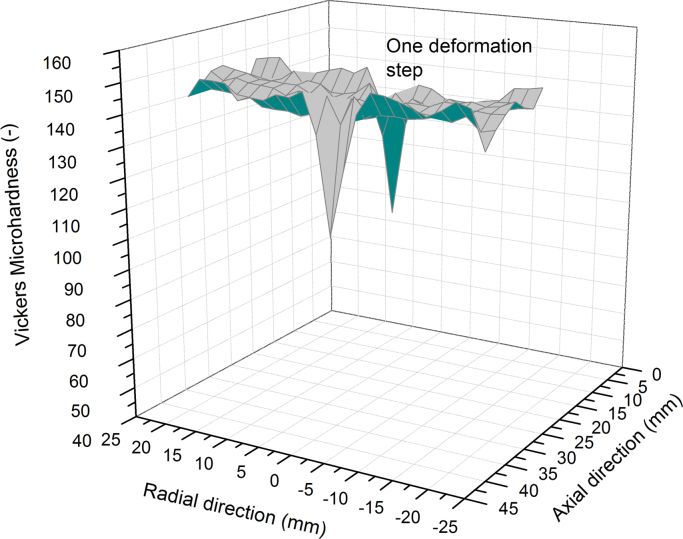
Hardness map along the radius and the axial direction measured for the specimen deformed only with one single deformation step.

A reasonable explanation for the slight decrease in hardness along the axial direction may be based on a temperature rise during the deformation process. It is generally known that SPD-processed structures exhibit a rather poor thermal stability compared to coarser grained materials [14,15]. In addition, for conventional HPT it is well documented that even when there is a massive heat loss through the large HPT-anvils there is still a temperature rise during deformation [16,17], which will also occur using IHPT. The increase may reach levels that lead to a static or dynamic recovery processes. The deformation zone acts as a heat source and is shifted after every single deformation step. Therefore, the first deformed areas, see Fig. 4e, are more strongly microstructurally affected than areas deformed towards the end of the process, Fig. 4d, because of the longer exposure to the generated heat in the deformation zone. To prove this assumption hardness measurements on the first sample, which was deformed with one single deformation step, were performed and compared with the second sample that was incrementally deformed up to 9 deformation steps, Fig. 5. The hardness results of the first specimen are presented in Fig. 6.

The maximum hardness in the singly deformed specimen is around the same level as measured on the incrementally deformed specimen regarding the area that was deformed in the last deformation step, see Fig. 5. In addition, the hardness level of the specimen in Fig. 6 is higher compared to the hardness measured in the area where the first deformation step of the incrementally deformed specimen occurred. This gives a strong indication that the hardness continuously decreased along the axial direction due to excess heat, Fig. 5. Therefore, in the future it is significant to cool the deformation tool in order to suppress annealing processes. Another useful strategy is to keep the deformation speed as low as possible. In the presented study one full turn had a duration of 15 min, which makes the time for the entire deformation process very long considering the high number of turns required for each deformation step. Therefore, it will be more useful to cool the deformation tool which would allow for a higher rotational speed.

A comprehensive inspection of the hardness distribution in Fig. 5 also shows a slight radial inhomogeneity of the hardness. This could principally be a cause of an applied deformation strain which is too low for saturation. A typical v.Mises strain, *ε*_*vM*_, for saturation in the case of pure metals is around 16–32 [18], which can be calculated with

(1) $$\varepsilon =\frac{2\pi r}{t\sqrt{3}}n$$

When one assumes a minimum strain, *ε*_*vM*_, of 16 and an approximate thickness, *t*, of the deformation zone of about 8 mm and 15 rotations as the number of rotations, *n*, the critical strain is reached at a radius, *r*, of approximately 2.5 mm. Hence, from this viewpoint saturation should be expected, which is not the case for IHPT presented here. A more likely reason for the gradient in the radial direction is connected to a strain-rate sensitivity known for UFG-materials [19]. The strain rate increases with the radius of the cylinder and may lead to a strain rate and thus radius dependent saturation hardness in the processed sample.

A comparison with the maximum achievable hardness using conventional HPT with literature data is a very difficult task as it is widely known that the saturation hardness of the same metal and comparable purity can vary markedly due to the large influence of impurities on the grain boundary mobility and so on the grain size [18]. There are reports indicating for pure copper similar saturation hardness levels as reached here [20,21]. However, using pure copper from a different supplier with a slight different amount of impurities may also lead to higher saturation values when conventional HPT is in use [22].

The presented deformation technique IHPT (incremental high pressure torsion) represents a further severe plastic deformation technique with the intention to upscale the specimen size of bulk-nanostructured materials. Compared with continuous high pressure torsion (CHPT) IHPT exhibits a different deformation path [23] and the material increase of the deformed material is along the axial direction. Very similar to this novel technique, incremental equal channel angular extrusion [24] has been introduced, where the shear deformation process is subdivided into increments as well. In incremental ECAP the hydrostatic stress component is however strongly restricted by the flow stress of the used plunger material. As another process showing similarities to IHPT, torsion extrusion should be mentioned [25] and some distinctive differences to this technique are shortly noted. In IHPT the material does not experience pronounced extrusion deformation which markedly decreases the diameter of the specimen. In addition, quite similar to classical HPT comparable amounts of hydrostatic pressures only depending on the diameter and applied force can be adjusted. In torsion extrusion processes the pressure is restricted by the strength of the plunger and the hydrostatic pressure component is also negatively affected by friction between the cylinder walls and the billet. The high hydrostatic pressure component in IHPT will allow the deformation of high strength materials and this even at room-temperature as already performed with classical HPT. As a drawback the additional small support cylinders which define the local deformation zone have to be changed manually, but could in future also be replaced by a hydraulic system. The large advantage of the presented principle working with support cylinders is that existing HPT facilities can be adapted to IHPT with moderate effort.

To summarize, a new SPD method, called incremental high pressure torsion (IHPT) was introduced which combines the advantages of conventional HPT and overcomes the limitation of a restricted specimen thickness. As a case study, the process was performed with a pure copper specimen showing an ultrafine-grained microstructure in the entire deformed section of the specimen having a diameter of 50 mm and a thickness of 40 mm. Future work will focus on extending the process to materials having a higher saturation hardness, e.g. alloys, steels and composites. In addition a cooling system will be required preventing additional heating and so temperature rise of the specimen during deformation.

## Footnotes

1 Due to the charateristics of torsion deformation the hardness measurements recorded on a plane, which cuts through the center of the cylinder, can be regarded as representative for the entire volume element of the corresponding axial position.
